# Neurofibromatosis type 1 associated with hypophosphatemic osteomalacia due to hypersecretion of fibroblast growth factor 23: a case report

**DOI:** 10.1186/s13256-020-02381-1

**Published:** 2020-05-09

**Authors:** Takahiko Obo, Nobuyuki Koriyama, Akinori Tokito, Kazuma Ogiso, Yoshihiko Nishio

**Affiliations:** 1 Department of Diabetes and Endocrine Medicine, National Hospital Organization Kagoshima Medical Center, 8-1 Shiroyama-cho, Kagoshima, 892-0853 Japan; 2 Department of Diabetes and Endocrine Medicine, Kagoshima University Graduate School of Medicine and Dental Sciences, Kagoshima University, 8-35-1 Sakuragaoka, Kagoshima, 890-8520 Japan

**Keywords:** Fibroblast growth factor 23, Tumor-induced osteomalacia, Neurofibromatosis type 1, Hypophosphatemia, 25-hydroxyvitamin D_3_

## Abstract

**Background:**

Neurofibromatosis type 1 is characterized by multiple café au lait spots and cutaneous and plexiform neurofibromas, and is one of the most common autosomal dominant hereditary disorders caused by mutations of the neurofibromatosis type 1 tumor suppressor gene. Osteomalacia in neurofibromatosis type 1 is very rare and is characterized by later onset in adulthood. In humans, fibroblast growth factor 23, which is a causative factor of tumor-induced osteomalacia, is not only a paracrine and autocrine factor, but is also a physiological regulator of phosphate balance in normal serum.

**Case presentation:**

Our patient was a 65-year-old Japanese woman whose neurofibromas began to appear when she was in elementary school. At age 28, she was diagnosed as having neurofibromatosis type 1. A spinal compression fracture and multiple rib fractures were identified in 2012 and 2017, respectively. Her laboratory findings revealed hypophosphatemia due to renal phosphate wasting and a high serum level of fibroblast growth factor 23. Neurofibromas located on the surface of her right forearm and left upper arm, in which a slight abnormal accumulation of tracers was observed on ^111^indium-pentetreotide scintigraphy, were surgically removed, but there was no improvement in hypophosphatemia or serum fibroblast growth factor 23 after surgery. Therefore, we administered eldecalcitol, which also failed to produce improvement in abnormal data. Subsequent combination with dibasic calcium phosphate hydrate led to improvement in some of the abnormalities, including hypophosphatemia. Immunohistochemical staining using anti-human fibroblast growth factor 23 antibody revealed slightly positive results, however, only one out of three amplifications of the fibroblast growth factor 23 gene was observed by real-time polymerase chain reaction, and no clear fibroblast growth factor 23 gene expression in the resected neurofibromas could be confirmed.

**Conclusions:**

We here describe a first rare case of a 65-year-old woman with neurofibromatosis type 1 associated with hypophosphatemic osteomalacia in which a high serum fibroblast growth factor 23 level was confirmed.

## Background

Neurofibromatosis type 1 (NF1) is characterized by multiple café au lait spots and cutaneous and plexiform neurofibromas (NFomas), and is one of the most common autosomal dominant hereditary disorders caused by mutations of the NF1 tumor suppressor gene (*NF1*) on chromosome 17 [1–3]. In addition, generalized skeletal abnormalities, such as mild short stature [4] and decreased bone mineral density (BMD) [5], are frequent in NF1. Osteomalacia in NF1, however, is very rare and is characterized by later onset in adulthood [6].

In humans, fibroblast growth factor 23 (FGF23), which is a causative factor of tumor-induced osteomalacia (TIO), is a 251 amino acid polypeptide hormone (32.5 kDa) belonging to the fibroblast growth factor (FGF) family [7]. FGF23 can be amplified from the human heart, liver, thyroid/parathyroid, intestine, lymph node, thymus, and skeletal muscle and bone by the reverse transcription-polymerase chain reaction technique [7–9]. Furthermore, it was reported that FGF23 acts on sodium–phosphorus co-transporter in the renal tubule and inhibits 1α-hydroxylation of 25-hydroxyvitamin D_3_ (25(OH)D_3_); thus, leading to renal phosphate leakage, hypophosphatemia, inappropriately normal or low 1α25-dihydroxyvitamin D_3_ (1α25(OH)_2_D_3_) levels, and decreased bone mineralization [10]. Hence, FGF23 is not only a paracrine and autocrine factor, but is also a physiological regulator of phosphate balance in normal serum [11].

Here, we report a rare case of a 65-year old woman with hypophosphatemic osteomalacia associated with NF1. Her serum FGF23 levels were elevated but no clear expression of FGF23 was confirmed in her surgically resected NFomas by immunohistochemical and molecular analysis.

## Case presentation

Our patient was a 65-year-old Japanese woman whose NFomas began to appear when she was in elementary school. She was born without any perinatal anomalies. At age 28, she was diagnosed as having NF1. In 2012, a spinal compression fracture was identified during a visit to a local orthopedic surgeon for lumbago. In 2017, she visited a local orthopedic surgeon with a chief complaint of lateral chest pain, and multiple rib fractures were identified. Hence, she was referred to our department for endocrinological examination. Pregabalin 50 mg was administered daily, and loxoprofen sodium hydrate 60 mg was used at the time of pain.

**Fig. 1:**
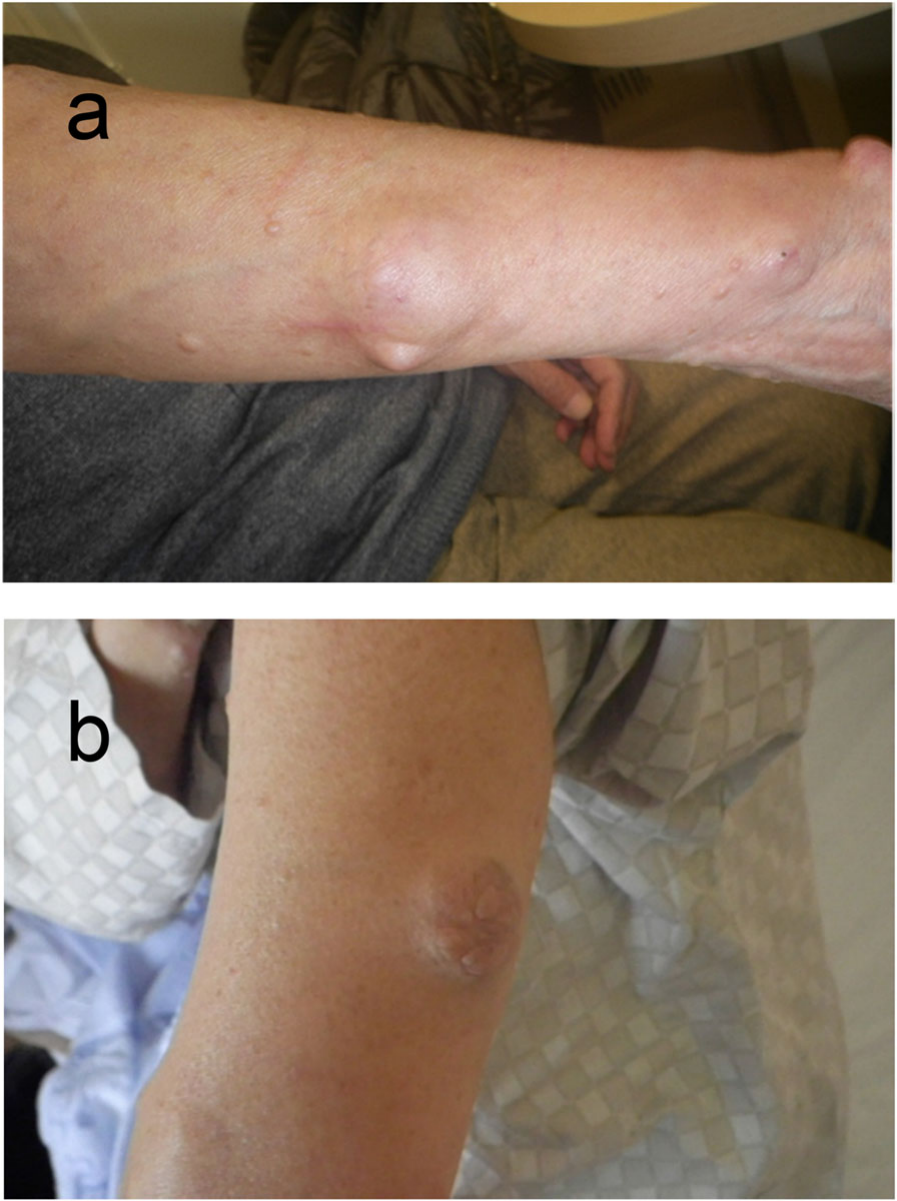
Photograph of the patient’s neurofibromas. Two neurofibromas were present: one on the surface of her right forearm (**a**) and one on the surface of her left upper arm (**b**)

**Fig. 2:**
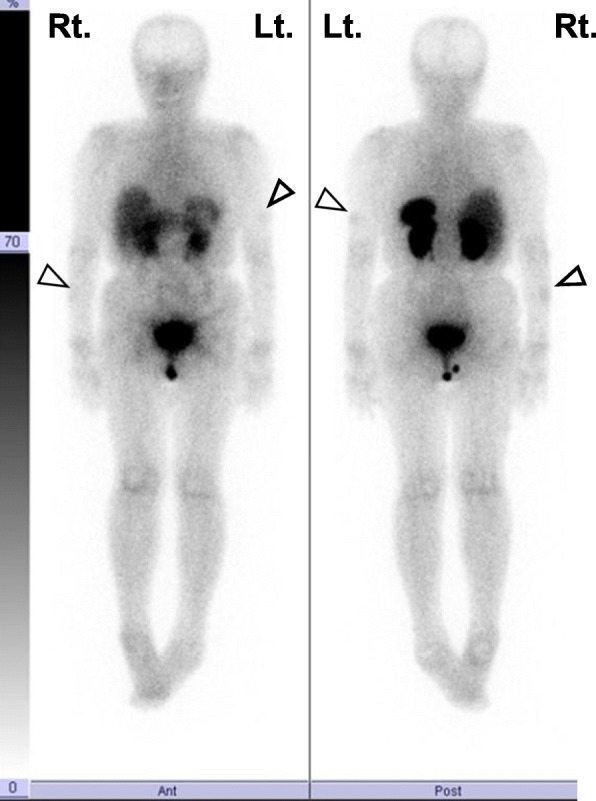
Octreoscan images. The *arrow heads* indicate light uptake into neurofibromas located on the surface of her right forearm and left upper arm. *Lt*. left, *Rt*. right

She was 147.1 cm tall, weighed 47.5 kg, body mass index was 22.0 kg/m^2^, body temperature was 36.6 °C, blood pressure was 105/72 mmHg, and pulse was 72 beats/minute and regular. She showed no mental retardation, and no pigmentation on her skin and oral mucosa. Her cardiopulmonary examination was normal. She had no abnormal abdominal and neurological findings or skeletal abnormalities. Soft NFomas of various sizes were scattered all over her body, and relatively large masses approximately 4 cm in diameter were present on the surface of her right forearm and left upper arm (Fig. 1). Her eldest daughter has also been diagnosed as having NF1. She was a caregiver; our patient drank alcohol occasionally but did not smoke tobacco. Her serum levels of inorganic phosphorus (IP), 25(OH)D_3_, and maximum transport of phosphorus in the renal proximal tubules (TmP/GFR) were inappropriately low (Table 1). Serum alkaline phosphatase (ALP), intact parathyroid hormone (intact PTH), bone-specific alkaline phosphatase (BAP), tartrate-resistant acid phosphatase 5b (TRACP 5b), and undercarboxylated osteocalcin (ucOC) levels were all elevated. Her serum level of FGF23 was high. The results of total blood cell count and other biochemical parameters were almost within normal limits (Table 1). BMD using dual-energy X-ray absorptiometry of the second to fourth lumbar vertebrae (L2–4, total) and left femoral neck were 0.764 g/cm^2^ and 0.504 g/cm^2^, with a young adult mean (YAM) of 64% and 54%, respectively. Computed tomography displayed no space occupying lesions other than NFomas on the body surface. Multiple areas of abnormal tracer uptake were seen in her rib on ^99^technetium (Tc)-methylene diphosphonate bone (MDPB) scintigraphy. Slight abnormal accumulation of tracers was observed in the NFomas located on the surface of her right forearm and left upper arm on ^111^indium-pentetreotide scintigraphy (Octreoscan) (Fig. 2). She did not agree with venous sampling because of difficulty in maintaining her supine position for prolonged periods because of systemic pain. Since she strongly desired resection of the NFomas on her right forearm and left upper arm, we respected her wish and excised them in February 2018. Pathology evaluation demonstrated benign NFomas. Unfortunately, there was no improvement in serum IP levels after surgery. Therefore, we administered eldecalcitol (active vitamin D_3_ analogue) 0.75 μg per day, which also failed to produce improvement in hypophosphatemia and other abnormal data. Subsequent combination with dibasic calcium phosphate hydrate (3.0 g/day) led to improvement in some of the abnormalities, including hypophosphatemia: IP, 3.1 mg/dL (2.7–4.6); ALP, 209 U/L (106–322); intact PTH, 46 pg/mL (10–65); BAP, 12.4 μg/L (3.8–22.6); and TRACP-5b, 309 mU/dL (120–420) (data not shown). After 6 months, serum calcium, IP, intact PTH, and BAP were 9.1 mg/dL, 3.6 mg/dL, 37 pg/mL, and 14.4 μg/L, respectively, and were stable in the normal range. Furthermore, pain also improved.

**Table 1:** Laboratory findings

Inspection Item				Reference range
Urine analysis	Protein	(−)		
	Glucose	(−)		
	Occult blood	(−)		
Urine biochemistry	TmP/GFR	1.77		22–40
Peripheral blood	WBC	3270	/μL	
	RBC	409 × 10^4^	/μL	
	Hb	11.5	g/dL	
	PLT	24.6 × 10^4^	/μL	
Biochemistry	AST	14	IU/L	
	ALT	11	IU/L	
	LDH	168	IU/L	
	ALP	641	IU/L	106–322
	γ-GTP	26	IU/L	
	T. Bil	0.95	mg/dL	
	Alb	4.24	g/dL	
	Na	144	mmol/L	
	K	3.4	mmol/L	3.6–4.8
	Cl	106	mmol/L	
	Ca	8.8	mg/dL	
	IP	1.9	mg/dL	2.7–4.6
	Mg	2.0	mg/dL	
	BUN	12.1	mg/dL	
	Cr	0.51	mg/dL	
	eGFR	90.4	mL/minute/1.73m^2^	
	FBG	97	mg/dL	
	HbA_1c_	5.2	%	
Endocrinology	Intact PTH	123	pg/mL	10–65
	25(OH)D_3_	14.0	ng/mL	20–60
	1α25(OH)2D_3_	57.2	pg/mL	20–60
	FGF23	57.0	pg/mL	< 30
	BAP	55.1	μg/L	3.8–22.6
	TRACP 5b	996	mU/dL	120–420
	ucOC	19.0	ng/mL	< 4.5

*Alb* albumin, *ALP* alkaline phosphatase, *ALP* alkaline phosphatase, *ALT* alanine aminotransferase, *AST* aspartate aminotransferase, *BAP* bone-specific alkaline phosphatase, *BUN* blood urea nitrogen, *Cr* creatinine, *eGFR* estimated glomerular filtration rate, *FBG* fasting blood glucose, *FGF23* fibroblast growth factor 23, *γ-GTP* γ-glutamyltransferase, *Hb* hemoglobin, *HbA*_*1c*_ glycosylated hemoglobin, *IP* inorganic phosphorus, *LDH* lactate dehydrogenase, *PLT* platelets, *PTH* parathyroid hormone, *RBC* red blood cells, *T*. *Bil* total bilirubin, *TmP/GFR* maximum transport of phosphate in the renal proximal tubules, *TRACP 5b* tartrate-resistant acid phosphatase 5b, *ucOC* undercarboxylated osteocalcin, *WBC* white blood cells, *1α25(OH)2D3* 1α25-dihydroxyvitamin D_3_, *25(OH)D*_*3*_ 25-hydroxyvitamin D_3_

**Fig. 3:**
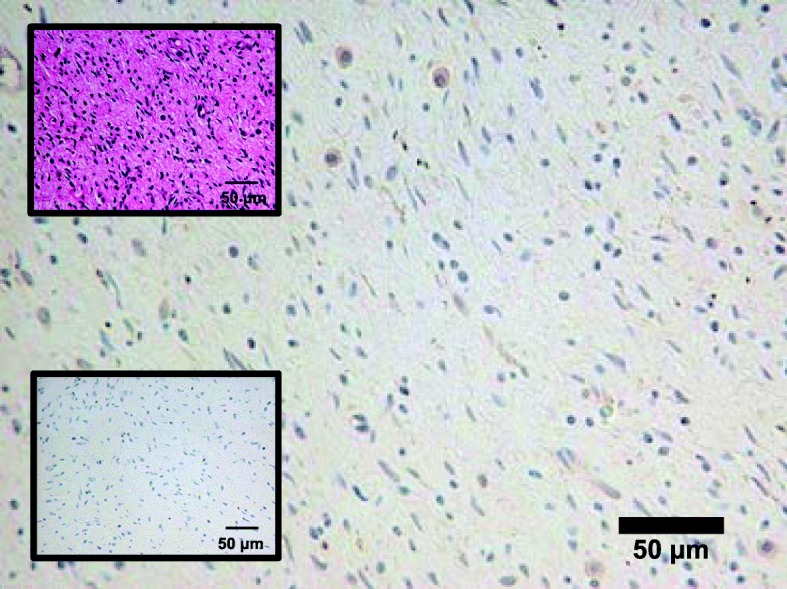
Immunohistochemical staining of fibroblast growth factor 23 in the resected neurofibromas. Single immunolabeling (peroxidase and diaminobenzidine tetrahydrochloride) of the resected neurofibromas. The *upper inset* shows hematoxylin and eosin staining. Ossified metaplasia, poorly differentiated foci of cartilage tissue, and osteoclast-like giant cells contained in many mesenchymal tumors are not observed, and dense proliferation of small short spindle-shaped cells against the background of hyaline or myxoma-like stroma are observed. The *lower inset* shows a negative control using normal rabbit immunoglobulin. The stromal cells in the tissue stained weakly positive using polyclonal rabbit anti-human fibroblast growth factor 23 antibodies

Immunohistochemical staining was performed on formalin-fixed and paraffin-embedded tissue from the resected NFomas, which demonstrated FGF23 weak positivity of the NFomas (Fig. 3). Pathological processing and evaluation was performed by GenoStaff Co., Ltd. (Tokyo, Japan).

**Fig. 4:**
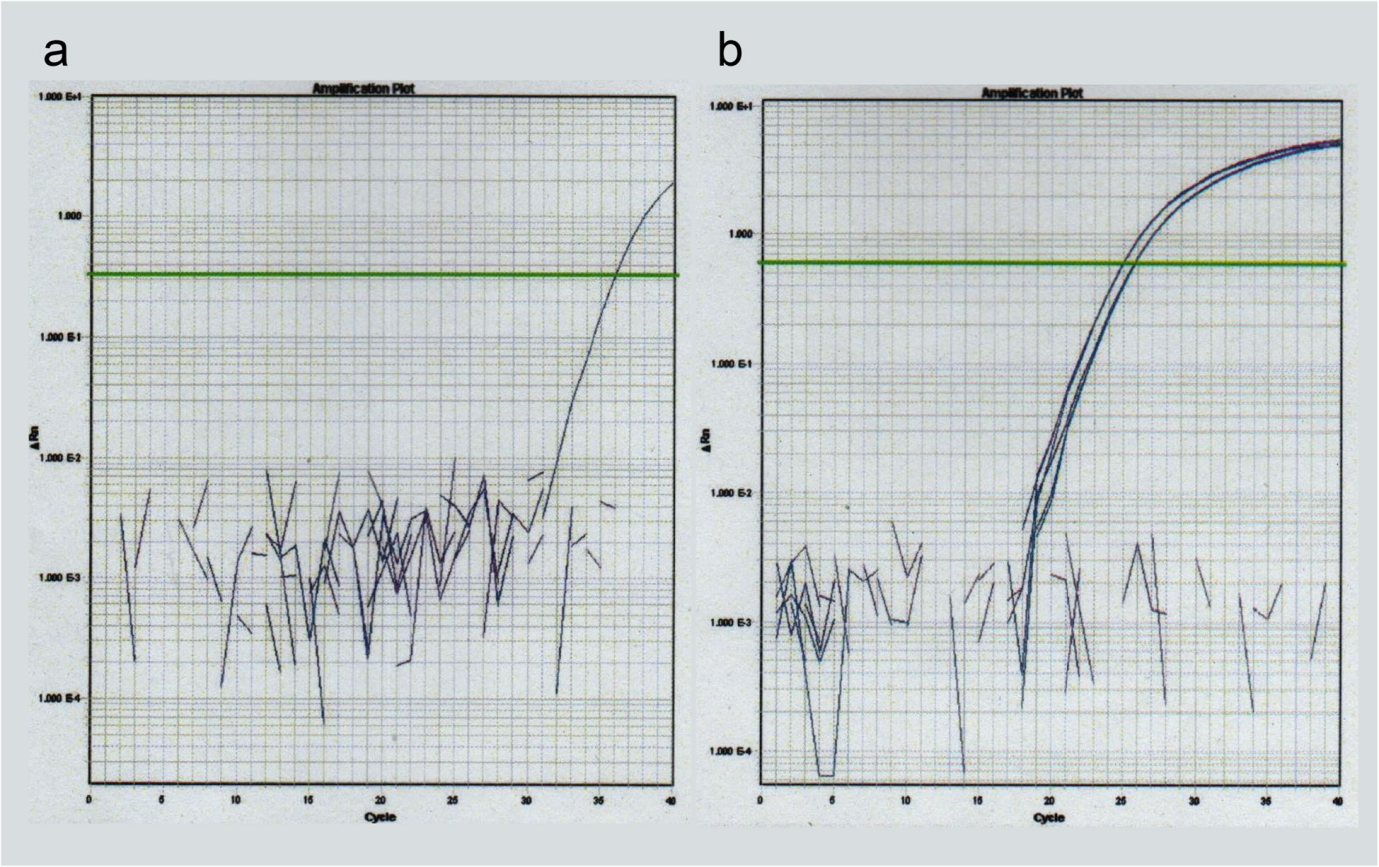
Fibroblast growth factor 23 gene expression analysis by real-time polymerase chain reaction in the resected neurofibromas. Amplification curve of fluorescence intensity. Amplification curves were drawn for the fibroblast growth factor 23 (**a**) and actin β (**b**) genes

Total ribonucleic acid (RNA) extraction from the formalin-fixed paraffin-embedded tissue samples was performed according to the manufacturer’s instructions. Human pancreas total RNA (Zyagen, San Diego, California, USA) was prepared as a control [7]. Next, we performed real-time polymerase chain reaction (RT-PCR) testing for housekeeping genes and actin β gene (*ACTB*), and the fibroblast growth factor 23 gene (*FGF23*), according to the manufacturers’ instructions. Amplification curve plotting using fluorescence intensity by ABI PRISM SDS 2.4 (Thermo Fisher Scientific Inc., USA) was performed (Fig. 4). All samples were amplified in triplicates. Once out of three times, the threshold cycle (C_T_) value for *FGF23* was 35.95 in resected NFomas, but it was not detected in human pancreas (Table 2). Unfortunately, these results did not clearly confirm expression of *FGF23* in the excised NFomas. These tests were conducted by GeneticLab Co., Ltd. (Sapporo, Japan).

**Table 2:** C_T_ value and mean C_T_ value of *FGF23* and *ACTB* by RT-PCR

Sample name	FGF23			ACTB		
	C_T_	mean C_T_	SD	C_T_	mean C_T_	SD
**Resected NFoma**	UD	–	–	25.7	25.76	0
	UD			25.8		
	36			25.8		
**Human pancreas**	UD	–	–	25	24.97	0
	UD			25		
	UD			25		

*ACTB* actin β gene, *C*_*T*_ threshold cycle, *FGF23* fibroblast growth factor 23 gene, NFoma neurofibroma, RT-PCR real-time polymerase chain reaction, SD standard deviation, UD undetermined

## Discussion and conclusions

The patient described here is the first case of NF1 associated with hypophosphatemic osteomalacia, in which a high serum FGF23 level was confirmed. Our patient was a 65-year-old woman diagnosed as having NF1 at age 28. Her laboratory findings revealed hypophosphatemia due to renal phosphate wasting and a high serum level of FGF23. Her NFomas located on the surface of her right forearm and left upper arm, in which a slight abnormal accumulation of tracers was observed on Octreoscan, were surgically removed, but there was no improvement in hypophosphatemia or serum FGF23 after surgery. Immunohistochemical staining using anti-human FGF23 antibody revealed slightly positive results; however, only one out of three amplifications of the *FGF23* gene was observed by RT-PCR, and no clear *FGF23* gene expression in the resected NFomas could be confirmed. We administered eldecalcitol combination with dibasic calcium phosphate hydrate, which led to improvement in some of the abnormalities, including hypophosphatemia.

TIO, also known as oncogenic hypophosphatemic osteomalacia, is a rare acquired paraneoplastic disease. TIO was first described by McCance in 1947 [12]. It is usually induced by benign mesenchymal tumors secreting excessive FGF23 [13]; in fact, FGF23 has been cloned as a causative factor of TIO [7]. Approximately 500 cases of TIO were reported worldwide up to 2018 [14]. On the other hand, osteomalacia associated with NF1 was first recognized by Gould in 1918 [15]. It is extremely rare, with fewer than 50 cases being reported [6, 16–21].

In our case, the typical biochemical pattern included low serum phosphate, increased phosphate excretion in urine with TmP/GFR reduction, and elevated ALP, BAP, TRACP 5b, and ucOC, indicating increased bone metabolism, along with elevated FGF23 concentrations and normal creatinine levels in serum (Table 1). We also observed elevation of serum intact PTH levels (Table 1). The serum levels of PTH are reportedly variable in TIO, although the reasons for these discrepancies remain unclear. Elevated levels of circulating FGF23 have been shown to promote the development of secondary hyperparathyroidism in predialysis patients through the suppression of 1α-hydroxylation of 25(OH)D_3_ [22], suggesting that excessive FGF23 might stimulate the parathyroid either directly or indirectly. Our patient’s 1α25(OH)_2_D_3_ levels were normal (Table 1). Since phosphate depletion stimulates renal 1α-hydroxylation of 25(OH)D_3_, resulting in elevation of serum 1α25(OH)_2_D_3_ concentrations, the normal level of 1α25(OH)_2_D_3_ in this case should actually be regarded as inappropriately low levels. Low values of 25(OH)D_3_ were also observed (Table 1). Low serum 25(OH)D_3_ concentrations, as seen in our patient, have been previously described in NF1 [17, 23].

In our patient, a slight increase in radiotracer uptake on Octreoscan (Fig. 2) was observed in the relatively large NFomas on the surface of her right forearm and left upper arm (Fig. 1). TIO-associated tumors express a series of somatostatin receptors (SSTRs) [24, 25], and Octreoscans reportedly effectively detect occult mesenchymal tumors [26]. In recent years, it has been recommended that entire body functional imaging tests, including SSTR imaging, should be conducted first for the localization of TIOs [15]. Our experience in this case showed that NFomas are likely to produce and secrete FGF23. Octreotide 50 μg, however, did not inhibit FGF23 until 8 hours after its administration (data not shown). According to a previous report, the role of somatostatin signaling in the causation of osteomalacia by phosphaturic mesenchymal tumors is unclear, and the efficacy of the somatostatin analogue in the treatment of patients with TIO is inconsistent [27].

A previous report on NF1-associated osteomalacia showed that hypophosphatemia improved after surgical resection of two large NFomas in a patient with neurofibromatosis [18]. When we provided this information to our patient, she wanted to remove her two large NFomas. Hence, we removed the two NFomas surgically, although it did not improve the hypophosphatemia. The mechanism behind hypophosphatemia in the setting of NF1 is not known. Only one case of NF1-associated hypophosphatemic osteomalacia, in which serum FGF23 was elevated, has been reported in the past, although immunohistochemical staining did not show FGF23 expression in the NFomas [21]. In our patient, immunohistochemical staining using anti-human FGF23 antibody revealed weak positive results (Fig. 3), but we could not prove *FGF23* expression in the resected NFomas by RT-PCR (Fig. 4 and Table 2). The reason why hypophosphatemia was not improved by excision of the NFomas is presumed to be continued production and secretion of FGF23 from FGF23-secreting tumor of unknown location. Reportedly, oral phosphate and vitamin D therapy is effective treatment for osteomalacia associated with NF1 [6, 18]. Hence, we administered eldecalcitol, although this, by itself, did not improve hypophosphatemia or other abnormal blood parameters, making it necessary to combine it with dibasic calcium phosphate hydrate. This also suggests that vitamin D deficiency is not the main cause of hypophosphatemia in NF1.

A limitation of our report is that we do not know why the increase in FGF23 was mild in our case. In a retrospective study of 144 cases of TIOs without NF1, however, cases with normal FGF23 levels (20.1 pg/mL) were also reported [28]. Our experience suggests that under hypophosphatemic conditions, normal to mildly high levels of FGF23 might need to be considered as obviously abnormal values. In addition, the possibility that a very small amount of FGF23 is synthesized and secreted from NFomas cannot be denied. A second limitation is that the possibility of increased production of FGF23 from osteocytes cannot be denied. Kamiya *et al.* reported that serum FGF23 levels showed a four-fold increase in NF1 conditional knockout mice (cKO) compared with age-matched controls, and immunohistochemistry showed significantly increased FGF23 protein in the cKO bones [29]. Further evaluations about this should be conducted in future. A third limitation is that lack of venous sampling has not completely ruled out the possibility of the presence of other tumors. A fourth limitation is that the possibility of genetic hypophosphatemic rickets could not be excluded in this case.

In conclusion, we reported a first rare case of NF1 associated with hypophosphatemic osteomalacia, in which a high serum FGF23 level was confirmed.

## Data Availability

Not applicable.

## Abbreviations

C_T_: Threshold cycle
L2–4: Second to fourth lumbar vertebrae
NF1: Neurofibromatosis type 1
NFomas: Neurofibromas
FGF: Fibroblast growth factor
FGF23: Fibroblast growth factor 23
*NF1*: Neurofibromatosis type 1 tumor suppressor gene
TIO: Tumor-induced osteomalacia
25(OH)D_3_: 25-hydroxyvitamin D_3_
1α25(OH)_2_D_3_: 1α25-dihydroxyvitamin D_3_
IP: Inorganic phosphorus
TmP/GFR: Maximum transport of phosphorus in the renal proximal tubules
ALP: Alkaline phosphatase
PTH: Parathyroid hormone
BAP: Bone-specific alkaline phosphatase
TRACP 5b: Tartrate-resistant acid phosphatase 5b
ucOC: Undercarboxylated osteocalcin
BMD: Bone mineral density
YAM: Young adult mean
MDPB: Methylene diphosphonate bone
Octreoscan: ^111^indium-pentetreotide scintigraphy
RT-PCR: Real-time polymerase chain reaction
*ACTB*: Actin β gene
*FGF23*: Fibroblast growth factor 23 gene
SSTRs: Somatostatin receptors
cKO: Conditional knockout mice
